# Family Support Experiences of Adult Persons with Intellectual Disability and Challenging Behaviour: A Scoping Review of Qualitative Studies

**DOI:** 10.3390/ijerph22060911

**Published:** 2025-06-07

**Authors:** Alice Nga Lai Kwong, Lisa Pau Le Low, Maggie Yat Cheung Wong

**Affiliations:** S.K. Yee School of Health Sciences, Saint Francis University, Hong Kong 999077, China; ycmwong@sfu.edu.hk

**Keywords:** intellectual disability, challenging behaviour, complex behaviour, family, experience, literature review

## Abstract

There has been scant research on the experiences of families caring for adults with intellectual disability who use challenging behaviour as a way of making their needs known. The aim of this scoping review was to synthesize the qualitative research data on the family support experiences of adult family members with intellectual disability who use challenging behaviour in this way. A systematic search was undertaken from five databases during December 2024 and updated in April 2025. A total of 20 studies were included in the review. Data were analysed using a thematic analysis method. The number of study participants in these studies ranged from 4 to 30. Most of them were parents and predominantly mothers. The results show that caring for adults with intellectual disability and challenging behaviour reflects a dual reality. The negative experiences refer to caregiving difficulties and inadequate formal support, while the positive experiences are associated with support from informal sources and caregivers’ fulfilment and gains. The available research did not offer sufficient data for a synthesis of how families might be affected by challenging behaviour. Future research should investigate how the negative and positive aspects of their families can be shaped to make a positive impact on caring for people with intellectual disability and co-existing challenging behaviour.

## 1. Introduction

The provision of family support for family members with intellectual disability has received increasing attention, particularly as people with intellectual disability are living longer, and, in many instances, can be expected to live in their family home well into middle age. In the context of human rights, intellectual disability is defined as a condition characterized by significant limitations in both intellectual functioning and adaptive behaviour, which originate before adulthood [1]. Challenging behaviour, also called complex behaviour, can be displayed in people with intellectual disability. Understanding the challenging behaviour presented by a person with intellectual disability depends on the context and characteristics of the behaviours. There are a range of challenging behaviours such as physical aggression or violence, disruptiveness, self-injury, and harassment. Other behaviours such as stereotypical behaviours and cultural or social norm-violating behaviours can also be labelled as challenging behaviours. These behaviours may persist throughout life due to ineffective strategies or a lack of support, and this can have serious disadvantages for adults with intellectual disability, such as interference with development, quality of life, social participation, and physical injury [2]. While the global prevalence of intellectual disability is approximately 1% [3], the prevalence of challenging behaviour in people with intellectual disability varies greatly in different populations ranging from 10 to 18% [2,4]. The determinants of challenging behaviour are multiple, involving a combination of biological, psychological, and social factors [5].

The role of family support in individuals with intellectual disability and co-existing challenging behaviour is crucial for fostering their participation in society and enhancing their overall quality of life. In the context of family support for adults with intellectual disability, the existing literature provides valuable insights into the broader needs and experiences of this population, even though it may not specifically address individuals exhibiting challenging behaviour. This broader literature serves as a foundation for understanding the complexities of family dynamics and support systems, which can be extrapolated to inform the experiences of families with members who may also exhibit challenging behaviour. While earlier studies have often emphasized the difficulties faced by family members who provide care [6,7,8,9], it is important to recognize the significant positive contributions that family involvement brings to the support experience. Family support is a long-term commitment that, despite its physical and psychological demands [10,11,12,13,14,15], provides opportunities for growth and connection within families [16,17,18,19,20]. Families play an essential role in providing physical care, assisting with daily living tasks, and nurturing their loved ones with intellectual disability. Although they may encounter challenges in managing unpredictable behaviours [15], these experiences can also lead to stronger family bonds and a deeper understanding of their loved ones’ needs. Research has increasingly highlighted the positive aspects of family support experiences, such as personal growth, the development of meaningful relationships, and a sense of role satisfaction. Care partners often find fulfilment in their ability to support their family members, which contributes to a greater sense of purpose and community [16,17,18,19,20]. Furthermore, studies have shown that factors like religiosity, spirituality, and both informal and formal support networks can enhance the quality of life for the family members of individuals with intellectual disability [16,17,18,19,20]. These positive influences underscore the importance of family involvement in creating a nurturing environment that benefits everyone involved.

There are differences in caregiving provision, services, and support for adults and children with intellectual disability and co-existing challenging behaviour [19,21]. While the existing literature has primarily focused on the experiences of families with young children with intellectual disability [22,23,24,25], there is a growing recognition of the need to explore the experiences of families with adults who have intellectual disability, particularly those exhibiting co-existing challenging behaviour. Our earlier works have laid the groundwork by examining the experiences of aging caregivers and their planning for the continued care of their family members with intellectual disability [26,27]. Difficulties and hardships were intensified owing to the loss of spouses and poor family relationships. Anxieties about future care arrangements, including the quality of this care and the involvement of other family members, were evident in these aging families [28]. These findings suggest that reliance on family and social support can alleviate caregiving stress and enhance emotional well-being. Despite the challenges posed by aging and the loss of family members, care partners often demonstrate remarkable resilience and employ positive coping strategies that help them navigate their support journeys.

Since the literature review by Griffith and Hastings 10 years ago [19], no subsequent reviews have addressed the experiences of the family support for individuals with intellectual disability and challenging behaviour. Therefore, a scoping review was conducted to identify the updated research in this area to promote a more balanced view of family support experiences in this population. Following a systematic approach, a scoping review is useful to examine the extent and nature of the evidence, summarize the findings, and identify the research gaps in a complex topic to plan and implement future research. The aim of this scoping review was to synthesize the qualitative research data on the family support experiences of adult members with intellectual disability using challenging behaviour and to identify any existing gaps in knowledge. The research question for this scoping review is “what does the evidence say on the experiences of caring for an adult family member with intellectual disability who also uses challenging behaviour to make their needs known?”.

## 2. Materials and Methods

The protocol of this scoping review is registered with the Open Science Framework (https://doi.org/10.17605/OSF.IO/6Q7AM). This review follows the Preferred Reporting Items for Systematic Reviews and meta-analyses reporting guidelines extended for scoping review (PRISMA-ScR), which is the most up-to-date and advanced approach for reporting scoping reviews [29,30].

### 2.1. Search Strategy

A systematic search was performed using the following databases: CINAHL, MEDLINE, Scopus, ProQuest, and Web of Science. Hand searching of reference lists of the searched papers was also performed. For the search terms, the term ‘intellectual disability’ was combined with the terms ‘challeng*’ and ‘behaviour’. These terms were combined separately (using AND as the Boolean operator) with the search terms ‘family’, ‘parent*’, ‘mother’, ‘father’, ‘sibling*’, ‘brother*’, ‘sister*’, ‘relative*’, ‘carer’ and ‘caregiver*’. A detailed outline of the database searches is provided in Supplementary File S1.

Articles were assessed against inclusion and exclusion criteria. Articles were included if the following inclusion criteria were met: (i) English full-text articles published in peer-reviewed journals between January 2014 and December 2024; (ii) studies using a qualitative approach or a mixed methods approach, where the qualitative data was reported on separately from the quantitative data; (iii) studies that explored family members’ experiences with caring for adults with intellectual disability who are above 18 years of age; and (iv) families whose members included mothers, fathers, and siblings of individuals with intellectual disability. Articles were excluded if they were (i) not qualitative research (i.e., quantitative research, systematic reviews, and meta-analyses); (ii) reporting mixed findings on non-family caregivers such as formal caregivers; (iii) not peer-reviewed publications; (iv) case studies, editorials, reviews, abstracts, or discussion papers; (v) studies targeting the caregivers of persons with intellectual disability who were below 18 years old; and (vi) studies focusing on formal or paid caregivers. The literature search was performed during December 2024 and updated in April 2025.

### 2.2. Study Selection

Two researchers (A.N.L.K. and L.P.L.L.) independently performed a two-stage review process. In the first stage, they screened all titles and abstracts based on the inclusion/exclusion criteria to identify potentially relevant papers. In the second stage, they selected the final studies eligible for inclusion by reviewing the full texts of the papers identified in the first stage. In the process, the reviewers discussed and made the necessary clarifications when there were inconsistencies in their decisions.

### 2.3. Data Extraction

Once the study selection was complete, data extraction was performed. Data on article characteristics (author, year of publication, country of origin, aim, setting, design, recruitment, methods of data collection and analysis, sample size, and participant characteristics) and data relating to the family support experiences of an adult with intellectual disability who also used challenging behaviour were extracted from the identified studies and charted using a table form.

### 2.4. Data Analysis

The data were analysed using the step-by-step guidelines for thematic analysis outlined by Braun and Clarke [31]. Initially, the reviewers read the findings of the included studies multiple times to become familiar with the data. Initial codes were created by coding the findings, after which these codes were organized into potential themes. The themes were then re-verified and cross-checked against the primary data and the coded extracts. Following this, similar themes were collected, grouped, and categorized. The analysis progressed by selecting extracts and relating the findings back to the research question. The reviewers discussed the results and updated the themes in an iterative process until consensus was reached.

## 3. Results

### 3.1. Search Results

**Figure 1:**
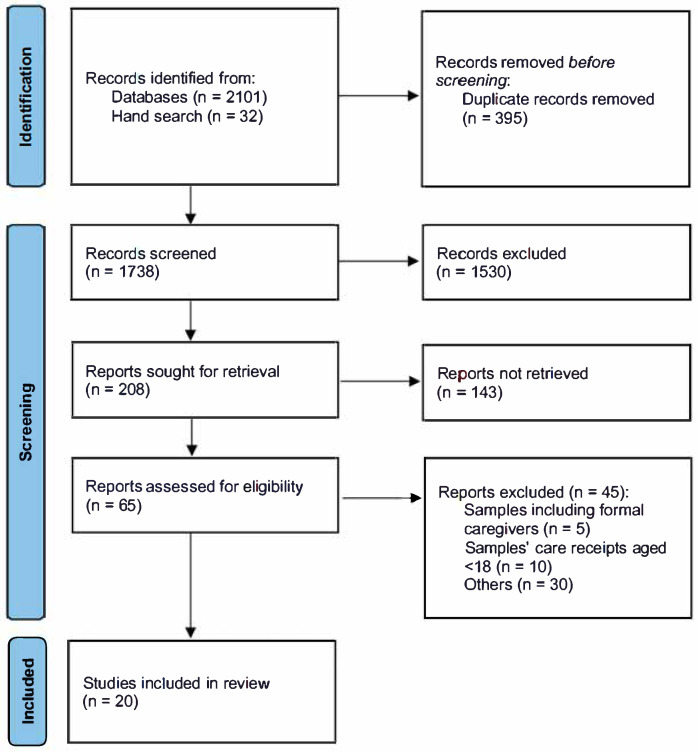
PRISMA flow diagram.

The initial search of the identified databases resulted in 2101 articles. An additional 32 articles were identified from the hand search. A total of 395 articles were rejected because they were duplicates. After a title screening of 1738 articles, 1530 were excluded according to the inclusion/exclusion criteria. Among the 208 articles screened, 143 were excluded because the samples did not meet the selection criteria. A total of 65 articles were then assessed from the full text and 45 were excluded because the samples and the findings did not meet the selection criteria. As a result, 20 studies were included in this review. The PRISMA flow diagram is shown in Figure 1.

### 3.2. Study Characteristics

The included studies varied by year, country, aim, research design, and samples. The studies were largely conducted in the United Kingdom (UK) (n = 6) and USA (n = 4). All the studies used a qualitative explorative design to examine the experiences with or perspectives on family support of an adult family member with intellectual disability who may also presents challenging behaviour. One study mainly investigated the parental experiences of children with intellectual disability and their transition to adulthood [32]. Another study investigated the family support experiences of people with intellectual disability receiving mental health services [33], and one study focused on future planning for adult children with intellectual disability [34]. Three studies explored the coping experiences of the family members of adults with intellectual disability during the COVID-19 lockdown [35,36,37]. Apart from four studies [7,38,39,40] that claimed to use convenience sampling, all the studies applied purposive sampling. While most studies employed semi-structured, in-depth, and individual interviews, only one chose focus group interviews [38] and one used a mixed approach including individual interviews and focus group interviews to collect data [41]. The methods of data analysis varied but thematic analysis was commonly used [33,34,35,37,40,41,42,43,44,45]. The number of family caregivers in these studies ranged from 4 to 30. Most of them were parents and were predominantly mothers. One study recruited only older fathers [46], one study was solely on older parents [47], and four studies were conducted on siblings [39,43,45,48]. One study [44] interviewed both caregivers and the family members of those with intellectual disability, but only the data from the family caregivers was extracted, analysed, and included in this review. Limited demographic information about the individuals with intellectual disability was provided, with only three studies supplementing information about the challenging behaviour of people with intellectual disability [7,35,37]. Seven studies reported the living arrangements of people with intellectual disability. Four studies included caregivers with a variation of residences, including a family home and a residential care facility [41,43,45,48]. The other four studies had a sample solely comprising individuals with intellectual disability living in the same household as their family [36,44,46]. Supplementary Table S1 shows the characteristics of the selected studies.

### 3.3. Themes

Three themes were formed to frame the family support experiences of adult persons with intellectual disability and co-existing challenging behaviour. These are as follows: caregiving difficulties, role as a key organizer of support services, and the positive aspects of caregiving (Supplementary Table S2).

#### 3.3.1. Caregiving Difficulties

As reported in all articles, caregiving difficulties appeared to be negative experiences commonly encountered by the family caregivers in the selected studies. The studies explored the experiences of parent caregivers in providing care for their child at different stages of adulthood. While parents wanted to provide less support to encourage independence once their son or daughter reached adulthood, they realized that their role of caring for their adult child with an intellectual disability was extended indefinitely due to the persistent and sometimes intensified challenging behaviours exhibited by their adult child with an intellectual disability [7,32,33,46,47]. The transition to adulthood for teenagers with intellectual disability was often a time of heightened uncertainty for parents. Parents in the study of Codd and Hewitt [32] were reported to experience many caregiving difficulties regarding the transition of their children with intellectual disability owing to the transition to another developmental stage and transitioning from child to adult services. Pryce and colleagues [44] described the fact that “the lives of caregivers had been consumed by their caregiving role” as they faced multifaceted restrictions in their personal, social, and working lives. These negative experiences could be the result of the changing health conditions of their adult child [37,42,44,47,49] and their adult child using challenging behaviour to present their needs [33,37,38,42,44]. The impact of caring included a lack of personal and leisure time, giving up work, unsatisfactory relationships with others, financial insecurity, and experiences of physical and mental health problems [7,36,37,38,46,49,50]. The COVID-19 pandemic further intensified these challenges, as family caregivers faced changes in daily routines and increased isolation, which contributed to escalating, difficult behaviours and a heightened unwillingness to cooperate on the part of their adult child, thereby complicating an already challenging caregiving situation [36,37]. Caregiving was further perceived as unpleasant because of societal stigma against their adult child with an intellectual disability. This negative perception would eventually lead to social isolation as parents had become reluctant to stay in contact with other families who did not have a good relationship with their child with an intellectual disability [37,47]. Older parents, who were experiencing the aging process while also fulfilling a caregiving role, experienced exacerbated uncertainty as they were worried about the care of their adult child with an intellectual disability once they could no longer be the main caregiver [35,38,42,47,49,50].

Four studies reported caregiving difficulties from the perspectives of the siblings of people with intellectual disability and co-existing challenging behaviour [39,43,45,48]. Caregiving for their adult brother or sister with an intellectual disability meant taking on additional family responsibilities. As described by Coyle and colleagues [48], the intergenerational transition of care is a unique feature of sibling support. Siblings’ responsibilities of support were changing due to aging parents, the aging sibling with an intellectual disability, an aging family network, as well as other family members’ unwillingness to take on the primary caregiving role which necessitated more intensive support. While they verbalized insufficient preparedness for the transition from the secondary to the primary caregiver role, their support was constrained by the structures, habits, and routines put in place by their parents who were previously the primary caregivers. As part of the adjustment to their caregiving role, they described their caregiving difficulties in terms of personal sacrifices, struggling with life choices, and inadequate support. They also reported the impacts of sibling caregiving on family cohesion and functioning. Siblings, as noted in the studies of Chase and McGill [39] and Yacoub et al. [45], described feelings of asymmetrical sibling relationships and neglect by parents. The siblings-in-law in Karni-Vizer et al.’s study [43] reported tension and difficulties in the couple relationship due to communication challenges and the behaviours exhibited by their partner’s sibling with an intellectual disability.

#### 3.3.2. Role as a Key Organizer of Support Services

As reported in 15 of the 20 articles, findings showed that family caregivers were the key organizers of support services and often advocated for the needs and rights of their family members with intellectual disability. A range of experiences highlighted the significant challenges posed by the management of challenging behaviour, which affected interactions with formal support services for adults with intellectual disability. Many caregivers described barriers to accessing and locating appropriate services [7,32,33,38,46,48], insufficient formal services [32,34,46,47], exclusion from needed services [38], and the poor quality of services [40,41]. Although some described positive experiences with formal services [41,42,50], these were largely negative experiences. These negative experiences were often exacerbated by the transition from children’s services to adult services, which families noted were frequently inadequate in addressing the complexities associated with challenging behaviour. Specifically, they reported significant frustrations with poor hospital care, limited community support, and a shortage of specialist services capable of effectively managing challenging behaviour. They also experienced a lack of coordination between services and clinicians, as well as impractical recommendations from professionals that did not adequately consider the behavioural challenges faced by their family members with intellectual disability [7,32,33,34,39,40,41]. The onset of the COVID-19 pandemic further intensified these challenges [35,36]. Families perceived formal care providers as lacking the necessary knowledge to care for patients with challenging behaviour, or that they just did not want to pass it on to the families [40]. While a few families appreciated virtual support from professionals through phone and video consultations, the majority reported receiving very limited assistance during the pandemic [36]. As families felt increasingly isolated from a formal support system [32,33,38,41,47], they often had to rely on informal support networks, such as parent support groups, and incurred the significant costs associated with private services when public options were unavailable or unsuitable [33]. The emotional toll of these negative experiences was substantial, with many caregivers linking their struggles to heightened anxiety and distress, particularly as they fought to secure appropriate support for managing challenging behaviour [32,40,46].

#### 3.3.3. Positive Aspects of Caregiving

A total of 13 out of the 20 studies identified the positive aspects of caregiving. Despite the negative aspects of caregiving, family caregivers’ positive caregiving experiences outweighed their negative experiences, and thereby helped them to cope and continue with the caregiving journey [39,46]. Caregivers’ positive experiences were associated with help and emotional support from informal sources such as other family members, extended families, parents, and families in similar situations [32,42,44,47]. Strengthened family relationships were also identified as a major positive aspect. While primary caregivers appreciated the support received in their lives that helped them cope with caregiving challenges, they emphasized the importance of family cohesiveness and their feeling of responsibility in maintaining positive family well-being [32,35,43,45]. The positive aspects of caregiving also concerned obtaining a sense of role identity. For example, primary caregivers perceived caring for their family members with intellectual disability as a natural and expected role. The parents of people with intellectual disability could integrate their roles of a carer and parent, which helped them accept that caregiving was meaningful to them. Such perception was explicit in the parent caregivers who lived together with their child with an intellectual disability under the same roof [44,46,50].

Another positive aspect was related to personal accomplishment and growth. Feeling satisfied and proud of the caregiving role, family caregivers discovered a purpose in life and gains in personal growth as a result of their caregiving role [7,44,45,50]. This growth was particularly evident for father caregivers in Dunn and colleagues’ study [46]. Caregiving facilitated the fathers’ re-evaluation of their life priorities. They placed more importance on the family unit and became aware that they had become a better and more caring individual through their caregiving experiences. Furthermore, they recognized their competence in the caregiving role, feeling proud of using their caregiving expertise to advocate for their son/daughter’s care. Similarly, siblings acknowledged the personal growth that occurred as a result of caregiving. They saw their caregiving experience as one of the defining features of their lives. In Chase and McGill’s study [39], the participants characterized themselves as more mature than their peers and attributed personal characteristics such as empathy, patience, acceptance, and independence to caring for a sibling with an intellectual disability who may have also had co-existing challenging behaviour.

## 4. Discussion

By presenting the characteristics and findings of a heterogeneous sample of qualitative studies across countries and regions, this scoping review provides a broad overview of the current research on family support experiences in the context of adults with intellectual disability who use challenging behaviour. This review examines the extent, nature, and gaps in the existing literature based on a wide perspective. Despite differences in culture, values, and health and social care services between the countries, this scoping review describes the common aspects of caring for adult persons with intellectual disability in order to provide health and social professionals with a broad, comprehensive, and diverse view of the phenomenon from the family perspective.

The synthesized data confirmed that caring for adult people with intellectual disability and co-existing challenging behaviour reflects a dual reality of both positive and negative experiences. Negative experiences refer to caregiving difficulties, highlighting several demands that are faced by the family members of an adult person with an intellectual disability who uses challenging behaviour. While some of these are also faced by parents whose children follow a typical developmental pathway, others are related to issues concerning the consequences of the aging problems in adult persons with intellectual disability such as dementia. Previous research shows that the level of caregiving difficulty is affected by the extent of public services and community support received by the family [51]. This review underscores that the family members of adults with intellectual disability report more negative experiences with healthcare and social services compared to those who care for children with similar disabilities. The concerns about insufficient support systems for adults highlight the difficulties families face in accessing necessary services, particularly as individuals transition out of paediatric care. The pervasive fear and uncertainty regarding their adult family member’s future emerges as a prominent finding, compounded by caregivers’ exhaustion from years of advocating for adequate services. However, the family members of adults with intellectual disability are generally tired of fighting for services as they have been worn down after a lifetime of advocating for these services. Additionally, these families express frustration with professionals, often citing experiences of insensitivity and limited understanding of challenging behaviour from formal service providers. This may stem from a general lack of awareness about the unique experiences and needs of families during the transition into adulthood for individuals with intellectual disabilities [20,52,53]. These insights point to the urgent need for improved support and information for families navigating support during this critical period.

In contrast to the negative findings, this review has identified several positive aspects of caregiving. The data synthesized in this review enhance our understanding of the family caregiving journey by moving beyond the emphasis on problems to emphasizing positive experiences in the context of caring for adult people with intellectual disability. A previous study, which compared quality of life between families with adults with intellectual disability and those with younger-aged children, demonstrates that families with a member older than 18 have a better quality of life [21]. Although there is no conclusive evidence on the reasons behind this, one explanation may be that families with older-aged members with intellectual disability have longer years of caregiving experience and thus, they have maintained a higher degree of stability throughout the caregiving journey. It could also be that people with intellectual disability can settle down as they age. As previously identified in this review, the positive aspects of caregiving for adult family members with intellectual disability include a strengthened family relationship, satisfaction with their role identity, personal accomplishment, personal growth, role competence, and a commitment to the family member with an intellectual disability. These findings correspond to Lloyd et al.’s [54] literature review suggesting that the positive aspects of caregiving can be conceptualized into eight categories: role satisfaction, emotional rewards, personal growth, competence and mastery, faith and spiritual growth, relationship gains, and a sense of duty and reciprocity; these positive caregiving aspects arise from the experiences gained from the caring itself and are derived from the dynamic between the caregiver and the care recipient.

The positive aspects of caregiving appear to be a positive appraisal response resulting from effective coping with a difficult caregiving situation [16,55]. Such a phenomenon could be understood as deriving positive meaning from the caregiving experiences by families with an older-aged member with an intellectual disability. According to the Meaning Making Model [56], an individual makes sense of the positive emotions and stress-related growth resulting from a stressful encounter. This involves an individual’s appraisal of a set of beliefs, goals, or purposes about the world and themselves, and the individual applies this meaning to those attached to stressful situations. The literature has demonstrated that family caregivers who have successfully found meaning in their caregiver role are more likely to cope well with the challenges they face and adjust to difficult caregiving situations [51,57,58]. Therefore, deriving positive meaning represents the strategies they use to manage the caregiving demands that are perceived as burdensome in caring for adults with intellectual disability. It has also been found to predict subjective quality of life among the family members of people with chronic illnesses [59].

The findings from this scoping review draw some policy and practice implications. The review highlights the importance of comprehensive support services tailored to the unique needs of families. Development of integrated service models that combine medical, psychological, and social support is crucial. Many families report feeling ill equipped to manage challenging behaviour due to a lack of knowledge and non-specific services or resources that target challenging behaviour. Public policy should prioritize funding for training initiatives by providing families with evidence-based strategies for support, to empower families and improve the quality of care provided to the families. Additionally, this review reveals that caregivers often need to create highly structured routines and environments to help mitigate challenging behaviour. This requirement can lead to a lack of spontaneity and restrict the family’s ability to engage in typical social activities. Access to respite care can give family caregivers breaks to reduce stress and prevent burnout.

This review has identified some research gaps. First, recent research on family experiences when caring for individuals with intellectual disability and co-existing challenging behaviour often overlooks the unique experiences posed by challenging behaviour. While studies typically highlight the overall support experiences of families with adults who have intellectual disability, they often do not delve into the nuanced impacts of challenging behaviour on family dynamics and well-being. This gap in the literature limits our understanding of how these complex behaviours specifically affect families’ physical, psychological, and social experiences. Addressing this oversight is crucial for developing targeted interventions and support systems that effectively respond to the unique needs of families navigating the complexities of challenging behaviour in their loved ones. Indeed, the existing literature on challenging behaviour has largely indicated that attempts by formal care providers to address challenging behaviour through the feasibility testing of training models works better in this context [60,61]. It must be acknowledged that what works for formal care providers in handling the challenging behaviour of persons with intellectual disability differs from what works for families. Having challenging behaviour in addition to an intellectual disability can further complicate family caregiving situations; thus, it is of utmost importance to understand the lives, needs, and support of these family caregivers, particularly those living in the same household with adult persons with intellectual disability. Furthermore, this review has revealed that there are no conclusive results on the actual process of how the family caregivers of persons with intellectual disability and challenging behaviour derive meaning from their support experiences in the literature. In other words, we still do not understand the mechanism of how family caregivers’ positive experiences can be enhanced so that they counterbalance the negative consequences of caregiving and empower caregivers to continue their role in caring for their family members. Future research will need to explore family caregivers’ perceptions of the negative and positive aspects of caregiving and how such meaning-making experiences of caring for a family member with an intellectual disability and co-existing challenging behaviour can be supported. Lastly, the family caregivers of the selected studies vary by country, region, and culture. They present a diverse cultural perspective, but the number and scope of related studies from non-Western countries have been limited. In this review, we found only one study that was conducted in Asia.

This review has some limitations. It includes a relatively small number of studies. Given the aim of the review—to explore family support experiences—only qualitative studies were included. Additionally, studies might have been excluded due to the decision to only include studies that reported on the caregiving of adult people with intellectual disability; thus, this might have led to the loss of important and valuable findings. However, a variety of databases were used that covered a broad area of fields including healthcare and social sciences, and the search terms covered diverse groups of family caregivers. The reference lists of the relevant empirical studies were also examined to identify other studies not captured by the database search. Another potential limitation concerns the language setting. As only English publications were included, some valuable studies may have been missed. Lastly, the implications for practice may be limited to specific cultural contexts. As different ethnic groups may respond to caregiving in different ways or may need distinct kinds of support, it is important to examine how cultural differences impact their caregiving experiences. A systematic review of a specific country or region with a similar cultural background can be considered in the future.

## 5. Conclusions

This scoping review has found that positive and negative experiences co-exist in the family caregivers of adult people with intellectual disability who also use challenging behaviour. Family caregivers feel suffering but, at the same time, they also find positive aspects in caregiving. It should be highlighted that caregivers’ dissatisfaction with formal support and services dominated their negative experiences. Public services need to make more of an effort to improve healthcare and social services by understanding the challenges the families of adults with intellectual disability and challenging behaviour have encountered. This review has confirmed that the recent research on family experiences when caring for individuals with intellectual disabilities often overlooks the unique experiences posed by challenging behaviour. For future research, the outcome of this review implies the need to explore family support experiences when managing challenging behaviour manifested by adult persons with intellectual disability. In addition, future research should investigate how the negative and positive aspects of caregiving can be shaped to derive positive meaning in caregiving for people with intellectual disability with the aim to empower families and improve the quality of care provided to the families.

## Data Availability

This study is a review of the publicly available literature and does not involve the generation of new data. All sources used in the review are cited in the reference list. No additional data are available.

## Supplementary Materials

The following supporting information can be downloaded at https://www.mdpi.com/article/10.3390/ijerph22060911/s1: File S1: Search strategy; Table S1: Summary of the reviewed studies; and Table S2: Caregiving experiences identified across the studies.
