# Supplementary material for: Family Support Experiences of Adult Persons with Intellectual Disability and Challenging Behaviour: A Scoping Review of Qualitative Studies
Source: Int J Environ Res Public Health. 2025 Jun 7;22(6):911. doi: 10.3390/ijerph22060911 (PMC12192965; doi:10.3390/ijerph22060911)
Supplement: Supplementary file 1 [file ijerph-22-00911-s001.zip › ijerph-3636100-supplementary.pdf]

Supplementary File S1. Search strategy

CINAHL

S1 intellectual disabilit* OR developmental disabilit*

S2 challeng*

S3 behaviour OR behavior

S4 S2 AND S3

S5 family OR parent OR mother OR father OR sibling* OR brother* OR sister* OR relative* OR care* OR caregiv*

S5 S1 AND S4 AND S5

MEDLINE

1 Exp intellectual disabilit*/ or exp developmental disabilit*

2 (challeng*) adj1 (behaviour or behavior). ti,ab,kf

3 family or parent or mother or father or sibling* or brother* or sister* or relative* or care* or caregiv*

1 and 2 and 3

Web of Science

#1 TS= "intellectual disabilit*" OR "developmental disabilit*"

#2 TS= "challeng* behaviour" OR "challeng* behavior"

#3 TS= family OR parent OR mother OR father OR sibling* OR brother* OR sister* OR relative* OR care* OR caregiv*

#1 and #2 and #3

Scopus

TITLE-ABS-KEY (“intellectual disabilit*”) OR TITLE-ABS-KEY (“developmental disabilit*”)

AND

TITLE-ABS-KEY (“challeng* behaviour”) OR TITLE-ABS-KEY (“challeng* behavior”)

AND

TITLE-ABS-KEY (“family”) OR TITLE-ABS-KEY (“parent”) OR TITLE-ABS-KEY (“mother”) OR TITLE-ABS-KEY (“father”) OR TITLE-ABS-KEY (“sibling*”) OR TITLE-ABS-KEY (“brother*”) OR TITLE-ABS-KEY (“sister*) OR TITLE-ABS-KEY (“relative*”) OR TITLE-ABS-KEY (“care*”) OR TITLE-ABS-KEY (“caregiv*”)

ProQuest

“intellectual disabilit*”OR “developmental disabilit*”

AND

“challeng* behaviour” OR “challeng* behavior”

AND

“family” OR “parent” OR “mother” OR “father” OR “sibling* OR “brother*” OR “sister* OR “relative*” OR “care*” OR “caregiv*”

Table S1. Summary of the reviewed studies.

	Author	Country	Year	Aim	Design	Number of participants (family caregivers)	Characteristics of family caregivers	Characteristics of adult persons with intellectual disability	Key findings
1	Banda et al.	USA	2024	To explore the financial concerns of mothers of adults with intellectual and developmental disabilities	• Convenience sampling<br>• Focus group<br>• Content analysis	5	• All mothers	Ages 20-40 years	Five themes:<br>1 Job-related difficulties.<br>2 Living expenses.<br>3 Access to essential services.<br>4 Skills deficits and/or challenging behaviour.<br>5 Long-term care.
2	Casey et al. (2023)	Ireland	2023	To explore the perceptions and experiences of mothers of adults with intellectual disabilities in Ireland of providing support in decision-making	• Purposive sampling<br>• Individual and semi-structured interview<br>• Thematic analysis	7	• All mothers<br>• Ages 50+	6 females and 1 male	Three main themes:<br>1 Support: family, services and routine.<br>2 Decision-making: importance, ability and Capacity Act.<br>3 Challenges: conflict, time, complex and enduring support needs and vulnerability.
3	Chase & McGill	UK	2019	To investigate the effects of having a sibling with a disability and behaviour described as challenging from	• Convenience sampling<br>• Semi-structured interview	6	• All siblings<br>• Ages 22-53 years	5 lived in residential care/supported living, 1 lived with	Four major themes:<br>1 Personal impact: they reflected their career choices, personal characteristics and

				adult siblings' perspectives.	• Interpretative phenomenological analysis			the family	struggles of living with a sibling with a disability and behaviour that challenges.<br>2 Relationships: they described an asymmetrical sibling relationship, and described their family as either close or distant.<br>3 Responsibilities: they described acquiring this responsibility as a choice, characterised themselves as "more mature" than their peers, reported a feeling of impending additional responsibilities for their brother or sister seeing themselves as the next-in-line carer.
4	Codd & Hewitt	UK	2021	To explore the lived experiences of ten parents who had a son or daughter with intellectual disability transition to adulthood (aged 18–25 years) with an intellectual disability.	• Purposive sampling<br>• Semi-structured interview<br>• Interpretive phenomenological analysis	10	• 7 mothers and 3 fathers<br>• ages 40–65 years (mean: 54.1).	Ages 18–23 years (mean: 19.8)	Three major themes:<br>1 Transition: The emotional experiences parents reported regarding their son/daughter's transition, including experiences of statutory services and reflections on the parent role.<br>2 Striving for Independence: parents' views of their son/daughter's independence and the

									process of separation and moving on.<br><br>3 Supporting the Supporters: how parents feel they are supported in their role, including the influence of the family context and family identity, and accessing support from wider systems discussing the importance of connecting with parents and families in similar situations.
5	Coyle et al.	USA	2014	To explore the transition in care from the perspective of a sibling who has replaced parents as the primary carer for an individual aging with intellectual or developmental disabilities.	• Purposive sampling<br>• Semi-structured interview<br>• Constant comparative qualitative analysis	15	• Sibling<br>• Ages 46-67 (mean: 57)<br>• 60% female	Mean age: 55	Three major themes:<br><br>1 Aging processes permeate the sibling carer role: The onset of dementia creates a new adjustment process, Age-related changes affect the ability of siblings with intellectual disability to age in place, Aging of the family network affects carer adjustment.<br>2 Planning shapes adjustment to the carer role. Negotiating the transition is impacted by parents' planning, Planning continues as a key component of the

									sibling care providing role.<br><br>3 Support received from other siblings impacts adjustment to the carer role, Formal support from service providers is helpful but may be difficult to negotiate.
6	Dreyfus & Dowse	Australia	2020	To explore what parents made about their day-today actions in life with their family member with intellectual disability and challenging behaviour.	• Convenience sampling<br>• Semi-structured interview<br>• Process type analysis	26	• All are parents<br>• 91% mothers	• Mean age 23<br>• 75% were males.	Parents enacted a range of complex and sometimes extreme activities across a variety of life domains. Parents spoke about: managing relationships with services; educating themselves and others; seeking support; resisting poor service delivery; assisting others; and making both small and significant changes.
7	Dunn et al.	UK	2019	To gain a more in-depth understanding of older father carers' experiences of parenting.	• Purposive sampling<br>• Semi-structured interview<br>• Constructivist grounded theory	7	• All fathers<br>• Mean age: 63.9 years).	• Ages 28-37 years<br>• 6 out of 7 lived in the same house with participants	Three conceptual categories:<br><br>1 Wearing different hats: how fathers' sense of identity had altered over the years.<br><br>2 Family comes first: importance placed on the family unit. Fathers re-evaluated their priorities

									and found a new identity in their parenting role.<br><br>3 Getting on in years: the challenges faced by ageing fathers parenting their son/daughter. While fathers admitted that there had been periods of extreme stress and that their other offspring had perhaps received less attention, most emphasized that the positives had far outweighed the negatives.
8	Gutowska	Poland	2022	To explore the situation of caregivers to adults with intellectual disabilities.	• Purposive sampling<br>• Individual and semi-structured interviews<br>• Content analysis	12	• Most were mothers (n=9)<br>• Ages 51-82	• Ages 20-49	13 thematic categories and subcategories were identified, along with illustrative examples. The main categories concerned everyday functioning, health, uncertainty, relationships with others, feelings, time, and the macro level.
9	Hamedanchi et al.	Iran	2016	To understand the unpleasant and bitter experiences of the elderly parents of children with intellectual disability.	• Purposive sampling<br>• In-depth interviews<br>• Descriptive phenomenology approach.	10	• Parents (5 males, 5 females)<br>• Ages 60–72.	• Participating in rehabilitation and training measures at the Comprehensive Rehabilitation Center.	“Bitterness” is one of the four emergent themes extracted in this study which has five theme clusters:<br><br>1 Experience of inappropriate behaviour toward the child with intellectual disability in the society.

									2 Experience of the society's failure to support the child with intellectual disability.<br>3 Sorrows experienced by the parents of persons with intellectual disability.<br>4 Experience of the child's problems<br>5 Barriers experienced in the care of the child with intellectual disability.
10	Ion & Lightfoot	USA	2023	To explore how family caregivers planned for their adult children's future caregiving needs in Romania, a country with a limited array of disability services	• Purposive sampling<br>• Individual and semi-structured interviews<br>• Thematic analysis	30	• 25 mothers and 5 fathers<br>• Ages 40–86	• Ages 18-49	Three categories:<br>1 Challenges faced by adults with intellectual disabilities and their caregivers: lack of services, exclusion and inaccessibility, no formal caregiving support, small or no social networks, overwhelmed and exhausted.<br>2 Caregivers' perceptions of future planning: planning is necessary, no help with planning, worries about the future, avoiding thinking the future<br>3 Engagement with planning: no planning at all, engaged in planning

									but no plan in place, had a formal plan
11	Irazabal et al.	Spain	2016	To investigate the impact on families of care as perceived by the main carers (mothers) of adult children with intellectual disability and mental disorders, and to learn about the experiences and opinions concerning the respite service.	• Purposive sampling,<br>• In-depth and individual interviews<br>• Content analysis	4	• Mothers	• Have associated mental disorders, who are linked to the center-based services and have attended respite services	Two categories:<br>1 'Family impact of care': there are various elements that generate a negative impact, but there are also elements that represent a positive impact on care.<br>2 'Respite service': All of the interviewed mothers agreed that respite was a valuable resource for their children but they spoke of the difficulty in making the decision to send their children to respite services.
12	Karni-Vizer et al.	Israel	2023	To examine how siblings and siblings-in-law of persons with intellectual disabilities experience the establishment of and processes within the couple relationship.	• Purposive sampling<br>• Semi-structured interviews<br>• Thematic analysis	24	• Six of the siblings of persons with intellectual disabilities were male, and six were female (ages 29-64).<br>• The group of siblings-in-law comprised seven women. and five men (ages 26–58)	• Ages 25-71	Three major themes:<br>1 Factors supporting establishment of the couple relationship<br>2 Tension and difficulties in the couple relationship attributed to being a sibling of a person with intellectual disabilities<br>3 Contribution of being a sibling to a person with intellectual disabilities to the couple relationship

13	Man & Kangas	Australia	2020	To examine carer experiences with mental health services for individuals with dual disabilities.	• Purposive sampling<br>• Semi-structured, in-depth interview<br>• Thematic data analysis	9	• Seven females, two males<br>• Ages 45-75, (Mean: 54.25).	• Ages 18-39 years (mean: 25.78).	Six major themes:<br>1 Service access and impact on carer coping: They reported difficulties in accessing appropriate services and highlighted the importance of collaboration and adjustments to suit individual needs of their child.<br>2 Degree of collaboration: Carers described positive and negative collaborative relationships with service providers and clinicians.<br>3 Positive impact of service provision: They reflected positive experiences of services.<br>4 Operational and resource limitations: Carers described negative experiences with services in relation to service delivery and provision, which was captured by two sub-themes.<br>5 Accommodating individual needs: Carers described the importance of service flexibility and willingness to accommodate their son/daughter and their needs as carers. Both

									positive and negative experiences were reported.<br>6 Limited service expertise: Carers described experiencing limited expertise in dual disabilities from a variety of services and professionals.
14	McKenzie et al.	UK	2017	To investigate the views of family carers about the support that their adult children with an intellectual disability had received in relation to their behaviour that challenged.	• Purposive sampling<br>• 3 semi-structured interviews and 1 focus group<br>• Inductive thematic analysis	8	No information	Ages 25-37	The study had a focus on positive behavioural support (PBS). Four major themes were identified:<br><br>1 The bedrock of support: All of the participants highlighted that good support required to be underpinned by a set of principles and values.<br><br>2 The impact of care: This theme explored what poor care felt like for the family carers and the impact of this on their child and themselves.<br><br>3 “It’s just been a battle”: The recognition that care was provided in a system, and that this system was not always experienced as supportive, to families or staff.

									4 “He’s my son”: This theme recognised the ongoing involvement of participants in their children’s lives, the emotional impact of having a child with intellectual disability and the importance of feeling that their children were now being supported in a positive and appropriate way.
15	Patel et al.	UK	2021	To gain insight into the ways parents of adults with ID coped during the first 2020 lockdown period.	• Purposive sampling<br>• Semi-structured interviews<br>• Thematic analysis	8	• 7 mothers and 1 father.	• Ages 18-43, diagnosed with Autism	Four major themes:<br><br>1 Powerless and unappreciated: Parents reported to have felt powerless and did not have as much in control during lockdown as they were before lockdown.<br>2 Coping under lockdown: They reported that many of their current caring responsibilities had already been routine before lockdown and that making sacrifices and putting their son/daughter first was what they had been used to.<br>3 Support: Parents reported that during the lockdown period they experienced a lack of support and communication from

									statutory services and many felt they had been left to cope alone.<br><br>4 The impact of lockdown on well-being: Lockdown was said by parents to have had negative as well as positive effects on their son's/daughter's well-being.
16	Pryce et al.	UK	2017	To explore the experiences of both older parents and adults with intellectual disabilities about being a lifelong caregiver/care recipient and their perceptions of the future.	• Purposive sampling<br>• Semi-structured interviews<br>• Explanatory thematic analysis	9	• Six mothers and three fathers<br>• Ages 65-85 (mean:76)	• Two males and one female<br>• Mean age: 45 years (range 38–57)	"Tolerating uncertainty" represents how parents and individuals with intellectual disabilities attempted to manage their anxieties about the future from the early years to the present day.<br><br>Six sub-themes:<br>1 Accepting the parenting role.<br><br>2 Facing challenges.<br><br>3 Being supported/being isolated.<br><br>4 Positive meaning making.<br>5 Re-evaluating as time moves on.<br><br>6 Managing future thinking.

17	Roberts et al.	USA	2024	To explore the experience of accessing treatment for self-injurious behaviour in individuals with intellectual and developmental disabilities	• Convenience and snowball sampling<br>• Individual interview<br>• Thematic analysis	15	11 mothers, 1 father and 1 sister	• 9 males and 4 females	Six themes:<br>1 Caregiver Driven.<br>2 Importance of the Provider-Caregiver Relationship.<br>3 Beliefs and Perceptions about self-injurious behaviour.<br>4 Practical Constraints.<br>5 Knowledge Gaps.<br>6 Wait Until Crisis.
18	Wos et al.	Poland	2021	To explore the experiences of parents of adults with intellectual disability in relation to remote support provided by public support agencies	• Purposive sampling<br>• Individual interview	22	16 mothers and 6 fathers.	• Ages 23-50<br>• Moderate or severe intellectual disability	Six themes:<br>1 Parent as therapist (required of constant support, lack of time, fear of losing skills, difficulty in taking up a job, lack of independence, no variation of tasks, lack of professional rehabilitation equipment)<br>2 Organization difficulties (need for assistance, respite care, lack of support in daily duties, need for hybrid solutions)<br>3 Material problems (need for financial support, lack of money to purchase electronic devices,

									appearance of unexpected expenses<br><br>4 Lack of social contacts (missing friends, loss of opportunities to develop social skills, loneliness, low frequency of meetings)<br><br>5 Positive solutions (adapting tasks to the needs and possibilities, constant contact, possibility of video meetings)<br><br>6 Difficult behaviour (unwillingness to do tasks at home, aggressive behaviour, emotional agitation, refusal to cooperate)
19	Yacoub et al.	Ireland	2018	To explore the experiences of adults who have a sibling with autism spectrum disorder and intellectual disability with challenging behaviour	• Purposive sampling<br>• Individual interview<br>• Thematic analysis	11	All siblings	No information	Four themes:<br><br>1 Differences – gains and losses<br><br>2 Impact on upbringing<br><br>3 Impact on sibling relationships<br><br>4 Future directions<br><br>Findings highlighted that challenging behaviour can cause emotional losses and can impact upbringing and

									the sibling relationship. Despite the siblings making adjustments, they continue to have concerns for the future.
20	Yi et al.	Korea	2024	To explore caregiving stress experienced by parents of adult children with intellectual disabilities during the COVID-19 pandemic	• purposive and snowball sampling<br>• Individual interview<br>• Thematic analysis	19	• 18 mothers and 1 father<br>• Ages 47-77 (mean 57)	• 15 males and 4 females<br>• Ages 21-45<br>• Disability severity: severe (n=9), moderate (n=8), mild (n=2)	2 themes<br>1 Caregiving burden: a worsening physical toll, agony of replacing lost services, constant and unstoppable worry, tolerance wearing thin, and increased conflicts<br>2 Deteriorating health: declining physical health and suicidal ideation due to endless caregiving

Table S2. Caregiving experiences identified across the studies.

**Themes**	**Experiences identified**
Caregiving difficulties	• Changed health conditions of caregivers and the persons with intellectual disability (Casey et al., 2023; Gutowska, 2022; Hamedanchi et al., 2016; Irazabal et al., 2016; Pryce et al. 2017; Yi et al., 2024)<br>• Managing challenging behaviour (Banda et al., 2024; Casey et al., 2023; Dreyfus & Dowse, 2020; Karni-Vizer et al., 2014; Man & Kangas, 2020; Pryce et al. 2017; Roberts et al., 2024; Wos et al., 2021 Yacoub et al., 2018; Yi et al., 2024)<br>• Family member with intellectual disability experiencing transition to adulthood (Codd & Hewitt, 2021; Dunn et al. 2019; Hamedanchi et al., 2016; Patel et al. 2021)<br>• Aging of the family and long-term care (Banda et al., 2024; Casey et al., 2023; Coyle et al., 2014; Gutowska, 2022; Hamedanchi et al., 2016; Irazabal et al., 2016; Patel et al., 2021; Yacoub et al., 2018)<br>• Personal sacrifices (Banda et al., 2024; Casey et al., 2023; Chase & McGill, 2019; Dreyfus & Dowse, 2020; Dunn et al., 2019; Gutowska, 2022; Irazabal et al., 2016; Wos et al., 2021; Yacoub et al., 2018)<br>• Financial constraints (Banda et al., 2024; Dreyfus & Dowse, 2020; Wos et al., 2021)<br>• Asymmetrical sibling relationship (Chase & McGill, 2019; Yacoub et al., 2018)<br>• Restricted relationships with others (Gutowska, 2022; Wos et al., 2021; Yi et al., 2024)<br>• Negative feelings (Casey et al., 2023; Chase & McGill, 2019; Gutowska, 2022; Irazabal et al., 2016; Roberts et al., 2024; Yacoub et al., 2018; Yi et al., 2024)<br>• Intergenerational transition of care (Coyle et al., 2014)<br>• Inadequate support from the family (Chase & McGill, 2019; Coyle et al., 2014; Roberts et al., 2024;)<br>• Additional family responsibilities (Casey et al., 2023; Chase & McGill, 2019; Coyle et al., 2014; Karni-Vizer et al., 2014; Roberts et al., 2024; Yacoub et al., 2018)<br>• Society's stigma against people with intellectual disabilities (Hamedanchi et al., 2016; Roberts et al., 2024;)
Role as a key organizer of support services	• Insufficient formal services, difficulty in accessing and locating appropriate services, poor hospital care, limited community support and services, a shortage of specialist services, ineffective approaches to managing challenging behaviour, lack of coordination between services, impractical recommendations (Banda et al., 2024; Codd & Hewitt, 2021; Coyle et al., 2014; Dreyfus & Dowse, 2020; Dunn et al. 2019; Hamedanchi et al., 2016; Ion & Lightfoot, 2023; Man & Kangas, 2020; McKenzie et al., 2017; Roberts et al., 2024; Yacoub et al., 2018)<br>• Lack of support and communication from statutory services during the COVID-19 pandemic (Patel et al., 2021; Wos et al., 2021; Yi et al., 2024)

	• Seeking informal support or private services when public services were unavailable or unsuitable (Man & Kangas, 2020)<br>• Responsive and expertise services (Casey et al., 2023; Man & Kangas, 2020; Wos et al., 2021)<br>• Respite service was a valuable resource (Dreyfus & Dowse, 2020; Irazabal et al., 2016)
Positive aspects of caregiving	• Support from other family members, extended families, parents and families in similar situations (Casey et al., 2023; Codd & Hewitt, 2021; Hamedanchi et al., 2016; Karni-Vizer et al., 2014; Pryce et al. 2017)<br>• Assisting other families/ peers in similar circumstances (Dreyfus & Dowse, 2020)<br>• Strengthened family relationship (Codd & Hewitt, 2021; Karni-Vizer et al., 2014; Patel et al., 2021; Yacoub et al., 2018)<br>• A positive sense of role identity (Dunn et al. 2019; Pryce et al. 2017)<br>• Personal accomplishment and growth (Chase & McGill, 2019; Dreyfus & Dowse, 2020; Dunn et al. 2019; Irazabal et al., 2016; Pryce et al. 2017; Yacoub et al., 2018)
